# Methodology of Using Terrain Passability Maps for Planning the Movement of Troops and Navigation of Unmanned Ground Vehicles †

**DOI:** 10.3390/s21144682

**Published:** 2021-07-08

**Authors:** Wojciech Dawid, Krzysztof Pokonieczny

**Affiliations:** Faculty of Civil Engineering and Geodesy, Military University of Technology, 00-908 Warsaw, Poland; krzysztof.pokonieczny@wat.edu.pl

**Keywords:** maps of passability, pathfinding, UGV navigation, cross country movement

## Abstract

The determination of the route of movement is a key factor which enables navigation. In this article, the authors present the methodology of using different resolution terrain passability maps to generate graphs, which allow for the determination of the optimal route between two points. The routes are generated with the use of two commonly used pathfinding algorithms: Dijkstra’s and A-star. The proposed methodology allows for the determination of routes in various variants—A more secure route that avoids all terrain obstacles with a wide curve, or a shorter route, which is, however, more difficult to pass. In order to achieve that, two functions that modify the value of the index of passability (IOP), which is assigned to the primary fields that the passability map consists of, have been used. These functions have a β parameter that augments or reduces the impact of the applied function on IOP values. The paper also shows the possibilities of implementation of the methodology for the movement of single vehicles or unmanned ground vehicles (UGVs) by using detailed maps as well as for determining routes for large military operational units moving in a 1 km wide corridor. The obtained results show that the change in β value causes the change of a course of the route as expected and that Dijkstra’s algorithm is more stable and slightly faster than A-star. The area of application of the presented methodology is very wide because, except for planning the movement of unmanned ground vehicles or military units of different sizes, it can be used in crisis management, where the possibility of reaching the area outside the road network can be of key importance for the success of the salvage operation.

## 1. Introduction

While planning military operations, the use of terrain properties is one of the key elements that should be taken into account. The ancient Chinese philosopher Sun Tzu, in his book entitled “*The Art of War*”, dating from the Late Spring and Autumn Period (roughly 5th century BC), noted that “with regard to ground of this nature, be before the enemy in occupying the raised and sunny spots, and carefully guard your line of supplies. Then you will be able to fight with advantage”. After many centuries, although the military techniques have developed significantly, the influence of terrain on the conducted operations still remains crucial, which has been proven in previous studies [1,2]. These publications demonstrate that proper use of terrain properties allows one to gain advantage, which is quite often the element that enables one to ultimately win the battle. Land cover and formation elements have to be taken into account in order to plan the movement of both whole military units and single vehicles. Currently, in the armed forces of countries that are members of the NATO (the North Atlantic Treaty Organization), terrain evaluation and the related passability analysis are realised in compliance with the provisions of standardisation documents [3,4,5], whose aim is to uniformise, and thus to increase the possibilities of interoperability between troops within the alliance. Pursuant to the provisions of these documents, land is divided into three basic passability classes (GO, SLOW GO, and NO GO terrain). This division is based on the analysis and evaluation of the influence of various classes of land cover and formation (relief, vegetation, hydrography, developed land, transport infrastructure, etc.) on the traction possibilities of vehicles. This approach allows the commander to plan the so-called avenues of approach for own and enemy troops. If possible, those routes should not cross NO GO and SLOW GO areas. In current military practice, these avenues of approach are introduced to the map manually, so the main aim of the authors, as discussed herein, is to automate this process.

### 1.1. Related Works

Studies on terrain passability and accessibility have been conducted by numerous scientists in various research institutions. Their research projects focus both on the influence of single terrain factors, such as vegetation [6,7,8] and soil [9,10,11,12] on passability conditions.

Some of the studies also analyse the cumulative influence of all terrain elements on passability [13,14,15]. The publications that deserve special attention are those that present complete terrain passability models. In this respect, the NATO Reference Mobility Model (NRMM) is an important project related to Cross Country Movement. It was developed and validated in the 1960s and 1970s by U.S. Army Tank Automotive Research, Development, and Engineering Center (TARDEC) and Engineer Research and Development Center (ERDC). It has been revised and updated through the years. This model is used for modelling the movement possibilities for various types of military vehicles and it covers the whole area of operation planning, including: scenario, terrain, and vehicle data [16]. Detailed descriptions of the Next-Generation NRMM are provided in the final report [17,18]. This model is connected to several research projects on various aspects of Cross Country Movement. These include, among others, the publication [19] which describes a framework for a stochastic approach for vehicle mobility prediction over large regions for integration into a NRMM. On the other hand, a comparison of the NRMM with other models in terms of the ability of military vehicles to traverse soft soils was presented in [20]. An example analysis of the influence of weather on passability conditions is the publication [21], whose authors present the results of research on the passability of vehicles that move in areas covered with snow.

This paper is an elaboration of the previous studies, where the team from the Military University of Technology proposed a system for automated generation of terrain passability and accessibility maps [22]. The conducted research deals with their application with the use of various sources of spatial data [23], discussed the problems of accuracy [24,25] and the methods of cartographic visualisation of the resulting maps [26]. Apart from that, the authors proposed methodologies, conducted tests and analyses on the application of artificial neural networks (multi-layer perceptron [27] and Kohonen Self Organizing Map [28]) to generate maps. Besides the analyses of passability on the operational level, which brought results in the form of generalised maps in a small scale, the authors also proposed methods to develop high-resolution (detailed) passability models, which allow users to plan the movement of individual vehicles [29,30] of a specific type. All the studies quoted above focused on developing maps that would reflect the actual terrain situation as accurately as possible, depending on their intended application. The advantage of these studies was the opportunity to analyse the terrain to make decisions defining the optimal corridors of movement for troops. However, they had to be entered on the map manually by the operator based on the analysis of the obtained passability map. The research presented here is an elaboration on the studies conducted so far, as it refers to the modification (transformation) of previously developed models (maps) to a form that will enable to define the route in a fully automated mode, taking the passability conditions into account. Although it might seem that the field of application is rather a niche, in fact the issues of defining routes for the cross country movement of vehicles is a subject of vivid interest in the academic circles. The related literature review is presented in [31]. It shows that the main area of focus of previous studies was the determination of optimal routes for the movement of autonomous robots (UGV—Unmanned Ground Vehicle) [32]. The authors of [33] used aviation photography for this purpose. Article [34] illustrates the applicability of the method through experiments with an unmanned ground vehicle in both structured and unstructured environments. The methodologies for defining movement routes for robots are also presented in works [35,36,37,38]. In this respect, the results of experiments that show non-standard solutions are particularly interesting. They doubtlessly include the method of defining routes through the cooperation between UGV and UAV (Unmanned Aerial Vehicle) presented in [39] and the use of ant colony algorithms for defining routes [40,41,42] also in military applications [43]. Current research focuses on the autonomous movement of vehicles in a dynamic environment, full of terrain obstacles [44,45,46]. Understandably, scientists pay special attention to considering obstacles caused by terrain formation, hence a large number of publications include Digital Terrain Models in the presented methodologies [47,48,49,50].

This study focuses on determining the paths for military purposes. This is connected with the Military Unit Path Finding Problem (MUPFP) which is the problem of finding a path from a starting point to a destination where a military unit has to move, or be moved, safely whilst avoiding threats and obstacles and minimizing path cost in some digital representation of the actual terrain [51,52]. An attempt to solve this issue taking into account military conditions (enemy position, terrain conditions, etc.) was presented in papers [53,54], whose authors additionally present proprietary software that generates routes automatically. The problem of modelling terrain accessibility and defining access routes is commonly addressed in strategic computer games. Such study is presented in publications [55,56]. On the other hand, the problems of military route planning in battlefield simulation are described in [57]. It is also worth mentioning the research on automated determination of movement paths for small groups of soldiers in urban areas [58].

### 1.2. Research Purpose

The studies presented above focused both on developing advanced terrain passability models, which would reflect the actual field conditions as accurately as possible, and the automated determination of movement paths for various types of vehicles, in military and non-military applications. The research presented here complements the studies listed in the previous section. The main research question which the authors were confronted with was the presentation of the methodology to convert the passability model that was developed and presented by the authors in numerous publications (including [22,24,27,29,30]) as the basis for the automated generation of the optimal path. This will doubtlessly lead to improved functionality and usability of the developed model. The article presents the results concerning route determination in various configurations for military operational units as well as for determining routes for single vehicles or even UGVs by using detailed passability maps. Another purpose of the research was to find a method of route generation in two basic configurations: longer, avoiding all terrain obstacles, and shorter, but harder to overcome. It allows for a variant approach to the planning process and selecting a route that can be fully defined as “optimal” in certain conditions. The authors present the methodology for generating such routes, along with the conducted practical tests. The last, but not least important aim of the research was to perform tests of route generation with the use of the two most commonly applied search algorithms: A* [59] and Dijkstra’s [60]. The authors analysed and compared both the resulting routes and the time required to generate them.

The direct and fundamental aim of the conducted research is the development of methodologies and solutions, which allow to develop a kind of “navigation system” enabling the determination of optimal routes for cross country movement on the basis of generated passability maps. The presented solutions will be implemented in a geoportal used for visualisation of the terrain situation, passability maps, and routes generated on the basis of them. Taking advantage of the geoportal and web services will surely help users to plan actions connected with the cross country movement, which we deal with during military operations and crisis management. The research presented here is a continuation and extension of the conference paper [61].

## 2. Materials and Methods

The determination of optimal routes was based on the passability maps whose development was presented in publication [22]. These maps are based on the division of terrain into primary fields of the same area. Each primary field is assigned an index of passability (IOP) value, which tells us whether the field is easy or difficult to pass. This value is the basis for the determination of the optimal route between two points. Moreover, different pathfinding algorithms (Dijkstra’s and A star) were used and the IOP values were modified with the use of two functions that provide other variants of routes (shorter but harder to overcome and safer—easier to overcome and, in most cases, longer). The whole methodology will be described in detail in this section.

### 2.1. Area of Research

**Figure 1:**
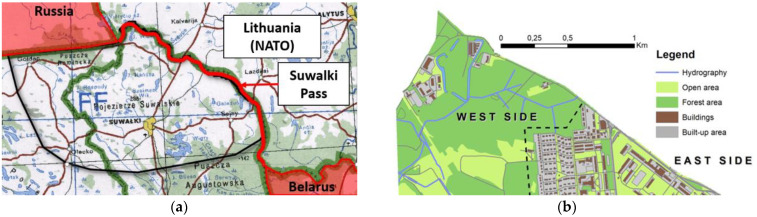
The location of the analyzed parts of the terrain. (**a**) Area near Suwalki city, (**b**) Area in Warsaw divided into two parts: urban (east side) and undeveloped (west side).

In order to show the universality of the presented methodology, the research was conducted on two kinds of terrain. The first area is located in the north-east corner of Poland near Suwalki city (54°06′ N, 22°56′ E) and there the determination of optimal routes was executed on the area of about 2500 square kilometers for the operational level of command. On the other hand, the second terrain is located in the west part of Warsaw (52°14′ N, 21°00′ E) and its surface area is only about 2 square kilometers. This analysis was conducted way more accurately on the basis of smaller primary fields (tactical level of command) and it was also enriched with the exclusion of impassable areas that came out of the microrelief analysis conducted with the use of the methodology that was widely described in [29] and of buildings. The location of areas of research is presented in Figure 1.

### 2.2. Description of the Developed Methodology

**Figure 2:**
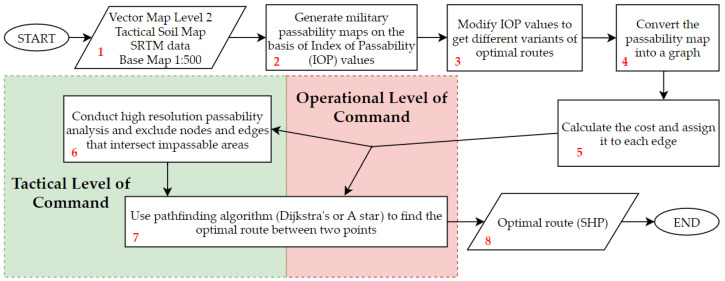
Block diagram of the optimal route determination process.

In order to show the whole methodology used to determine optimal routes in different configurations, a block diagram was created. It is presented in Figure 2 and the successive steps of the process will be described in detail afterwards.

The system developed by the authors [22], which has been enriched with an optimal route determination module and is now able to conduct the whole presented process. Its steps are described below.

#### 2.2.1. Block No. 1

**Figure 3:**
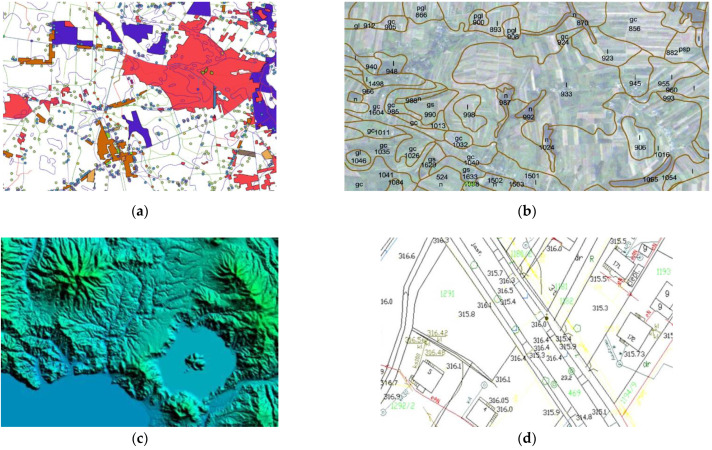
Examples of used data: (**a**) Vector Map Level 2; (**b**) Tactical soil map with extracted soil types; (**c**) Visualized SRTM data; (**d**) Base map in a scale of 1:500.

To create a military passability map we have to take into account multiple factors that influence terrain passability. Due to the fact that there is no comprehensive database that stores all necessary information, we have to use some of them (Figure 2, 1). The most important data source was Vector Map Level 2 data (VML2). It is a spatial database created as part of a national initiative that covers the whole territory of Poland and corresponds to a military topographic map in a scale of 1:50,000—M755 series [62]. The content of this product is described by the DIGEST (Digital Geographic Information Exchange Standard) scheme, which is a standard for digital geographic information exchange between different data users, data producers, and countries [63]. The database is grouped into 11 thematic categories and includes all types of land cover, such as hydrography, vegetation, cultural, transportation, industrial, etc. Overall, there are 206 feature classes in the database developed for the territory of Poland. An example of VML2 is presented in Figure 3a.

Another important factor is the type of soil that is crossed by troops. Their parameters are stored in the tactical soil map, which is a product released by the Military Geography Directorate in the form of SHP files. It is created on the basis of the soil-agricultural map in a scale of 1:100,000 and granulometric measurements of soils in Poland. However, the map does not take afforested areas into account. This product is designed mainly for conducting passability analysis of areas without a road network. An example of a tactical soil map is presented in Figure 3b.

Moreover, shuttle radar topographic mission data (SRTM) that are responsible for the elevation were applied to the system in a form of digital terrain model. These data are widely used in Polish Armed Forces and in the NATO countries. Their horizontal accuracy can reach about 30 m and vertical accuracy about 20 m. SRTM data are described accurately in publication [64] and they are visualized in Figure 3c.

On the other hand, in order to create a very accurate passability map, as in this research for the terrain in Warsaw, the base map in a scale of 1:500 was used. It contains the most detailed information that is available in Poland about all land cover elements such as the register of land and buildings, land development, and utility infrastructure. The map allows users to take account of all obstacles that may be located on a potential route. Its fragment is presented in Figure 3d.

#### 2.2.2. Block No. 2

Maps of passability are created automatically by a system developed by the authors that was described in [22] (Figure 2, 2). Their development is based on the division of terrain into primary fields of the same area. In the presented research, these fields are squares. Other shapes were not taken into account because their change does not cause significant differences in the final output, which was shown quite clearly in [24]. However, various sizes of primary fields allowed for obtaining different detailed passability maps. It is essential for the possibility to use these maps for planning operations and determining routes for military units of various level of command because, taking into account the width of the movement corridor defined in instruction [3], it is easy to set a size of the primary fields of which the passability map should be built. All sizes with the corresponding troop sizes are presented in Table 1.

In order to create a passability map, a value, which represents the possibility of overcoming this part of terrain, must be assigned to previously generated primary fields. This value is the index of passability (IOP), and it is computed on the basis of elements of land cover and landform (obtained from the data described in Section 2.2.1) that are located within the analyzed primary field. The index may take a continuous value from 0 to 1, where 0 indicates completely impassable terrain and 1 wholly passable terrain with excellent tractive properties. The more objects (e.g., rivers, lakes, or forests) hinder the movement, the bigger negative impact they have on the IOP and inversely, the more they facilitate the movement (roads, bridges, tunnels, or open areas without any objects), the higher the value of the IOP. Examples of passability maps in different resolutions are presented in Table 2. In this research, the authors focused on the analysis on high resolution passability maps (2 × 2 m, 3 × 3 m, 5 × 5 m, and 10 × 10 m). On the other hand, to show the universality of presented methodology, an analysis on low resolution map (1000 × 1000 m) was also included.

#### 2.2.3. Block No. 3

**Figure 4:**
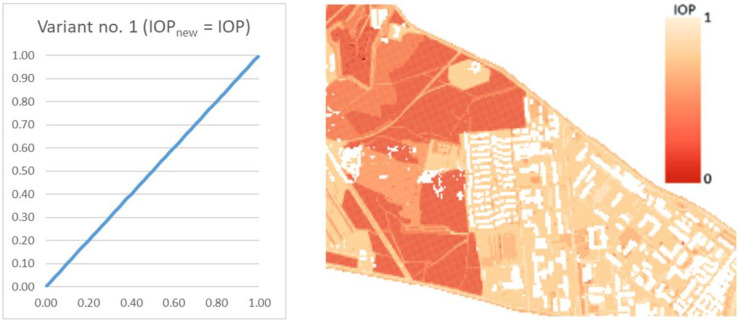
A chart of a function used in the starting variant and the corresponding passability map.

**Figure 5:**
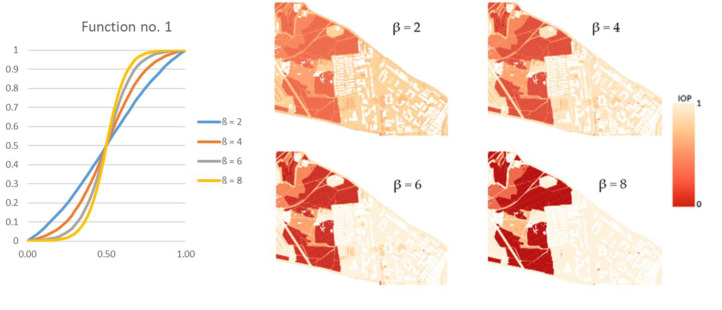
A chart of the function used in the second variant and the corresponding passability maps.

**Figure 6:**
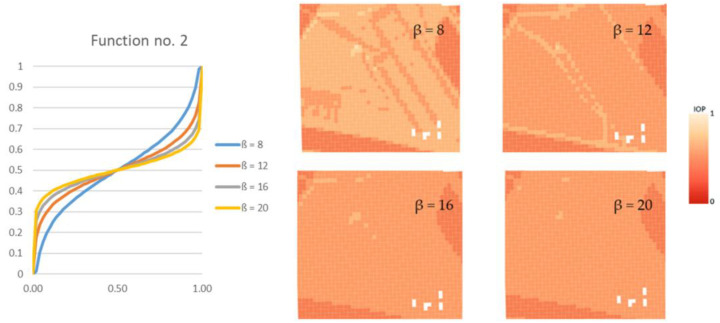
A chart of the function used in the third variant and the corresponding passability maps.

The crucial functionality of the developed system is the possibility to modify the IOP values in order to choose a variant of the route that is the most suitable for our needs (Figure 2 and Figure 3). As a result, three different variants of routes may be generated:

1. Finding the route directly from the computed IOP values. This variant may be called a starting variant because the index values are not modified before determining the route. This variant is presented below in Figure 4.
2. Finding the route for modified IOP values by means of determining their new values with use of the following equation:
  (1) $${\mathrm{IOP}}_{\mathrm{new}}=\frac{1}{1+e^{-\beta \cdot \mathrm{IOP}}},$$
  where β is a coefficient that amplifies or weakens the impact of the function on the IOP value, e is the Euler’s number (e ≈ 2.71) and IOP is an original IOP value. Tests were performed for a β parameter ranging from 2 to 18 because lower or greater values did not significantly change the new IOPs. The use of Equation (1) allowed us to augment the differences between impassable and passable areas, which are visualized in Figure 5. This variant enables to determine a route that goes through an easy to overcome terrain and in most cases, it is longer than the route in the starting variant.
3. Finding the route for modified IOP values by determination of their new values with use of the following equation:
  (2) $${\mathrm{IOP}}_{\mathrm{new}}=\frac{-\ln\left(\frac{1}{\mathrm{IOP}-1}\right)}{\beta +0.5},$$
  where ln marks the natural logarithm, IOP is an original IOP value, and the β parameter in this equation ranged from 2 to 24. Other values, similarly to the first function, did not significantly change the new IOPs. Thanks to this function, the IOPs distribution was flattened with a simultaneous decrease in the differences between passability indexes, which is presented in Figure 6. This solution enables to obtain a route which is significantly shorter but is leading through less passable areas.

#### 2.2.4. Block No. 3

**Figure 7:**
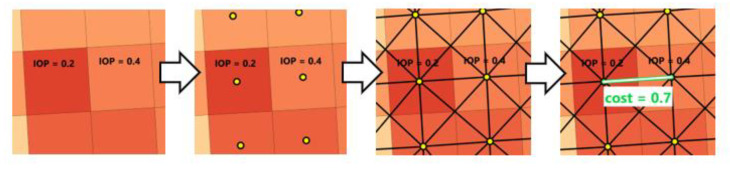
A way of generating a graph on the basis of the passability map.

The subsequent step of the process is the conversion of previously generated passability maps into a graph that consists of nodes and edges (Figure 2, 4). The methodology assumes that the centroids of primary fields are nodes and the connections between them are edges. On the basis of IOPs of neighboring primary fields, we are able to compute a cost value (Equation (3)) of the edge connecting the nodes that represent these fields. It is visualized in Figure 7.

#### 2.2.5. Block No. 5

Cost value will be used in the following steps to determine the optimal route (Figure 2, 5) and it is calculated with use of Equation (3):

(3) $$\mathrm{cost}\;=1-\left({\mathrm{IOP}}_{1}+\;{\mathrm{IOP}}_{2}\right)\;/\;2,$$

where IOP_1_ and IOP_2_ are values of neighboring nodes of the currently analyzed edge. The lower the IOP values are, the bigger the cost is, and the more likely it is that the pathfinding algorithm will not choose this edge as a part of the optimal route. All computed cost values are assigned to edges and the graph prepared in this way can then be used in further steps.

#### 2.2.6. Block No. 6

**Figure 8:**
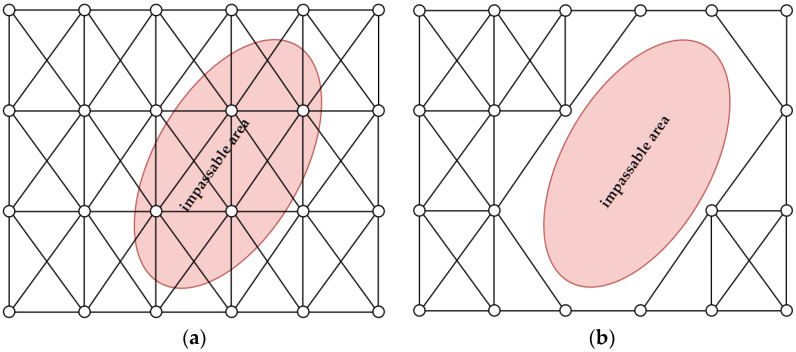
Exclusion of nodes and edges that intersect impassable areas: (**a**) Graph and impassable area before exclusion; (**b**) Graph after exclusion of nodes and edges.

This step is used in high resolution analysis (for the tactical level of command). It uses the methodology described in [29] to generate impassable areas based on digital elevation models (DEM) in high resolution (Figure 2, 6). Additionally, the authors assumed that buildings are objects that cannot be passed by a vehicle, so they were also considered as impassable terrain. Having obtained such areas we are able to find and exclude nodes and edges from the graph that intersect them. This step is visualized in Figure 8.

As a result of the exclusion, the number of edges to be processed in further steps decreases. As a consequence, the duration of the whole process is shortened. In this research, the determination of impassable areas was conducted for the 1 m DEM resolution because this value turned out to be optimal for this kind of analysis [29]. Moreover, all areas covered by building objects that are located within the analyzed terrain were also included as impassable ones. Subsequently, regardless of the resolution of the graphs, all nodes and edges that intersected impassable areas were deleted from the route determination process.

#### 2.2.7. Block No. 7

On the basis of the generated graph, we are able to determine the optimal route between two points (Figure 2, 7). In the research discussed here, this operation was executed with the use of two pathfinding algorithms: Dijkstra’s and A-star search algorithm. The first of them allows to find the shortest path in a graph with non-negative weights [60]. The use of this algorithm was presented in [65] where the authors demonstrated that the discussed algorithm managed to determine an optimal route between points randomly distributed within a city in Indonesia. Dijkstra’s algorithm realizes the so-called greedy approach. In each iteration, one of the unvisited nodes, which can be reached with the lowest cost, is chosen. After the determination of a path to a specific node, it will not be modified during the execution of the rest of the algorithm. Dijkstra’s algorithm is described in more detail in [66].

**Figure 9:**
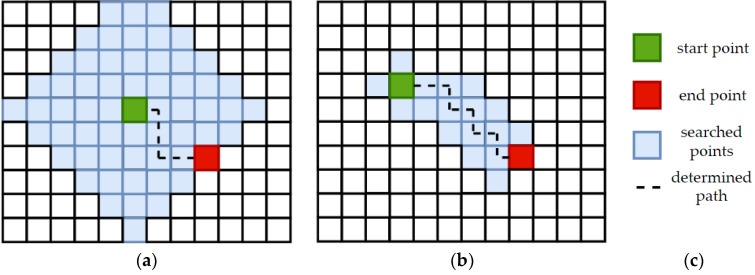
Comparison between the working principles of: (**a**) Dijkstra’s algorithm; (**b**) A-star algorithm; (**c**) Legend.

The second pathfinding algorithm used in this project was A-star. As opposed to Dijkstra’s algorithm, A-star performs an informed search. It takes into account the location of the source and target node and thanks to that the routing query prefers links which are closer to the target node. A more detailed description of A-star is presented in [67]. Moreover, this algorithm was used by some researchers. A team from Slovakia used A-star for planning a path for a mobile robot and showed that this algorithm was the fastest to find the path [68]. Esat Dere and Akif Durdu, researchers from Turkey, used the discussed algorithm to find the shortest path in transportation systems and provided some interesting results [69]. The comparison of these two pathfinding methods is presented in Figure 9.

#### 2.2.8. Block No. 8

**Figure 10:**
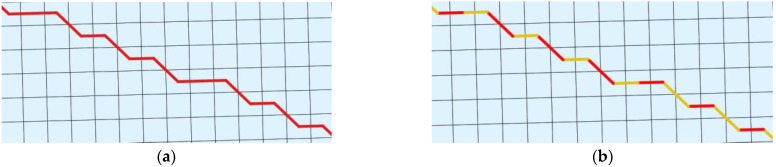
Difference between two kinds of output: (**a**) Whole route; (**b**) Segmented route.

The final step of the process is to export the determined routes to a shapefile (SHP) format (Figure 2, 8). Thanks to this we can distribute the output to different systems or platforms where it can be analyzed. What is more, the developed methodology allows us to generate two kinds of output: the whole and the segmented route. As a segment we understand a part of the whole route within a primary field. The difference is presented in Figure 10.

The whole route is the standard output while the segmented one can be used to monitor changes of terrain difficulties along the route in the form of a chart presenting e.g., the cumulative cost. All analyses are thoroughly discussed in Section 4.

## 3. Results

**Figure 11:**
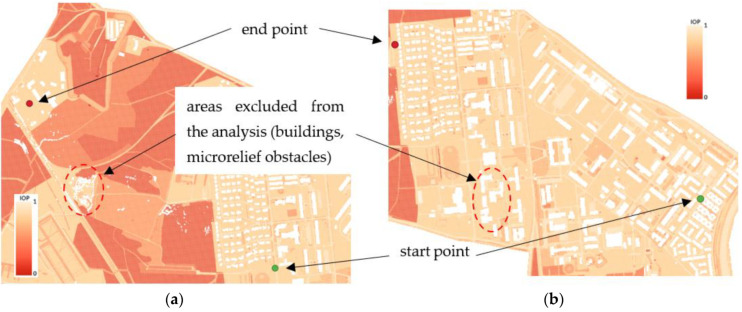
Start and end points of routes and areas excluded from the analysis (in white): (**a**) Undeveloped terrain; (**b**) Urban terrain.

Due to the fact that this article concerns the possibility of using the presented methodology in navigation of UGVs (very detailed level), the main attention will be focused on the results from high resolution passability maps (terrain in Warsaw). However, the analysis for the area near Suwalki city (operational level of command) better shows the dependences between used functions (described in Section 2.2.4), which will be presented in further sections. In the analysis, routes between start and end points were generated and they went through two types of terrain: urban and undeveloped. The location of the used start and end points is presented in Figure 11. In white there are also shown areas excluded from the analysis (buildings and microrelief obstacles), which should be avoided by generated routes.

**Figure 12:**
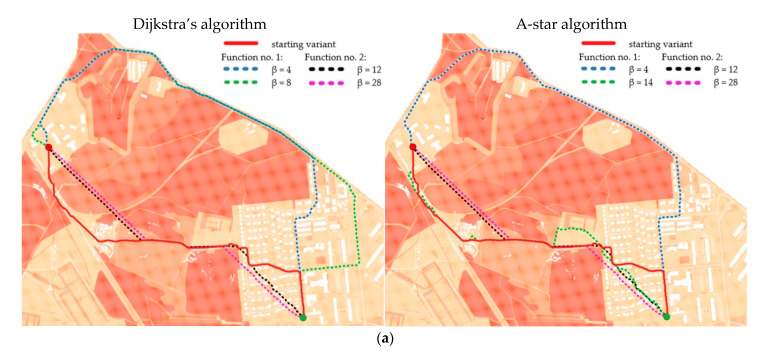

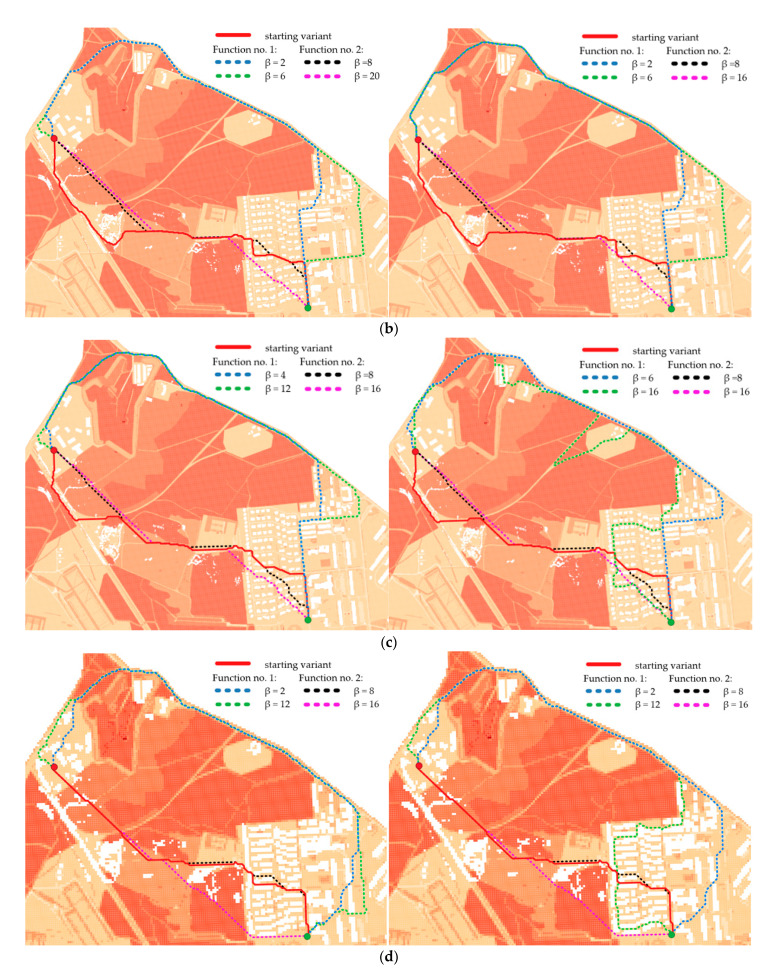
Optimal routes in the undeveloped area in different configurations and in the following resolutions: (**a**) 2 m; (**b**) 3 m; (**c**) 5 m; (**d**) 10 m.

**Figure 13:**
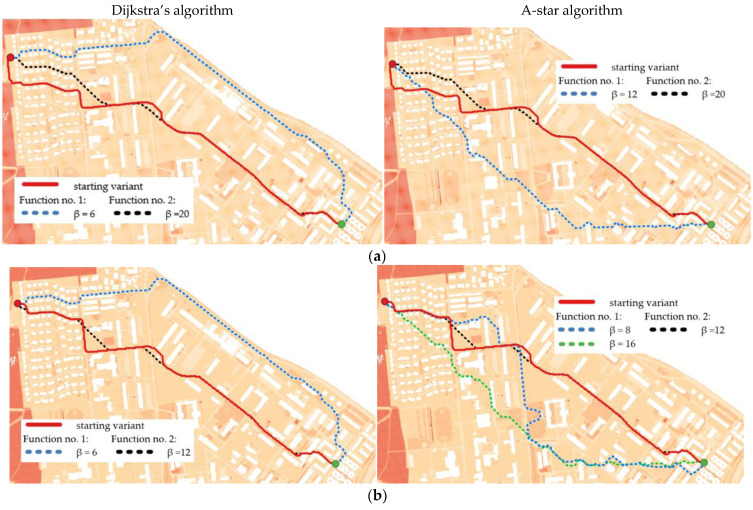

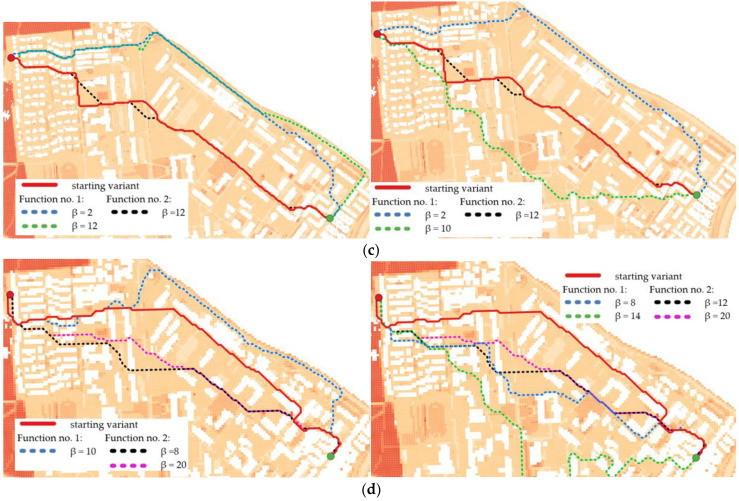
Optimal routes in urban area in different configurations and in the following resolutions: (**a**) 2 m; (**b**) 3 m; (**c**) 5 m; (**d**) 10 m.

**Figure 14:**
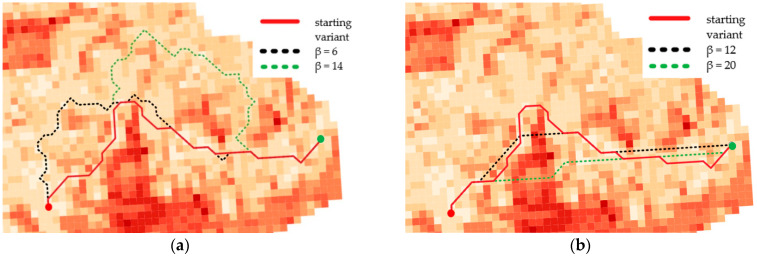
Optimal routes in the area near Suwalki city (operational level of command)—primary fields of 1000 × 1000 m size: (**a**) Function no. 1; (**b**) Function no. 2.

The illustrations below show the optimal routes generated for different functions and for various β coefficients. Their values were adjusted in order to show the changes in the determined routes. Different parts of the chosen terrain and consequently, different distributions of IOP values make the functions influence the road determination disparately. Figure 12 shows the results for routes in the undeveloped area, Figure 13 presents the outcome for routes in the urban area and the optimal routes for the area near Suwalki city are shown in Figure 14.

The generation times of routes are also a crucial factor in the analysis. They depend mainly on the resolution of the used passability map and slightly on the used algorithm. The whole process of generating a route was divided into two parts. The first one is the determination of a route and the second one is exporting the designated route to a Shapefile (SHP). Generation times on the example of a starting variant route in the undeveloped area are presented in Table 3 and the parameters of the server, on which all tests were carried out, are as follows:

- Model: DELL T640;
- Processor: 2 × Intel Xeon Gold 6230, 2.10 GHz;
- RAM: 64 GB;
- HDD: 4 × 1 TB SDD, RAID 5.

## 4. Discussion

**Figure 15:**
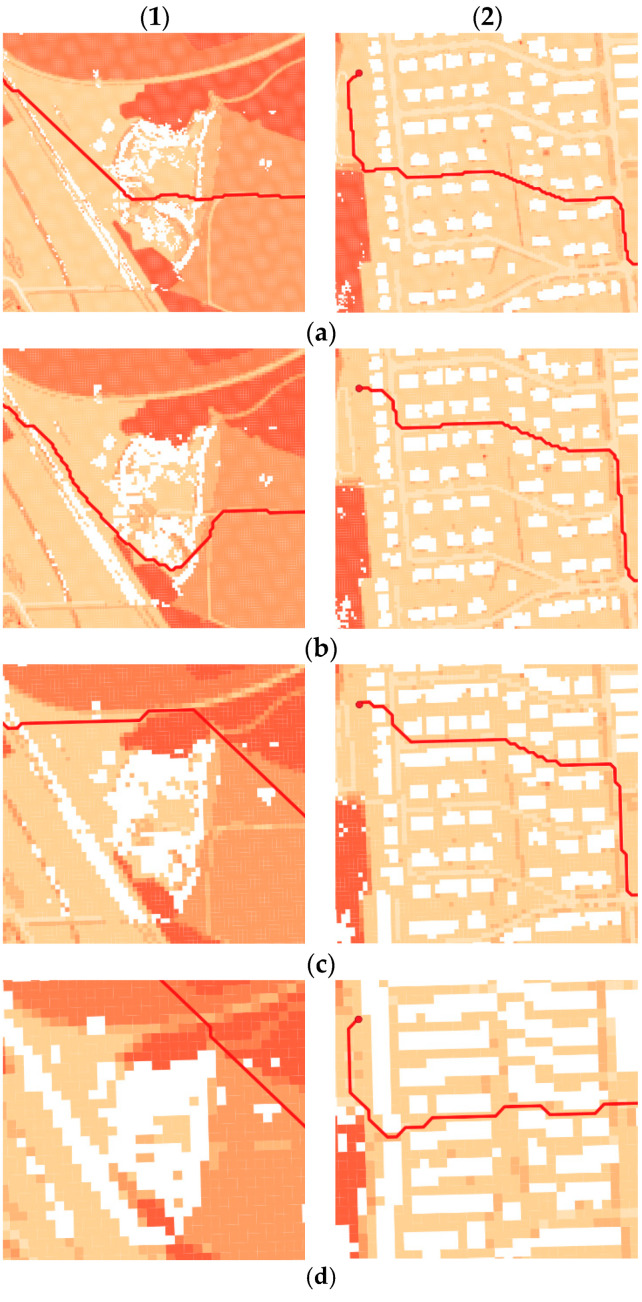
Change of the course of the starting variant route because of the generalization of passability map: (**1**) Avoiding a microrelief obstacle; (**2**) Avoiding buildings; (**a**) 2 m resolution; (**b**) 3 m resolution; (**c**) 5 m resolution; (**d**) 10 m resolution.

Generally speaking, the obtained results showed that the pathfinding algorithms used are able to determine optimal routes between two points. Optimal means that the routes were generated on the basis of a graph with weighted edges (cost values), which enabled us to choose the edges with the lowest cost. As a result of the exclusion of impassable areas obtained in microrelief analysis and buildings, unnecessary edges which were located on these parts of the terrain were removed from the graph. This operation ensures that the vehicle which will be moving along the determined route will not face an obstacle that will hinder or prevent the relocation. Different resolutions of the used passability maps, which were the basis for generating the graph, made the excluded areas more and more generalized, which forced the pathfinding algorithm to change the course of the route in some places. Such situations are presented in Figure 15.

**Figure 16:**
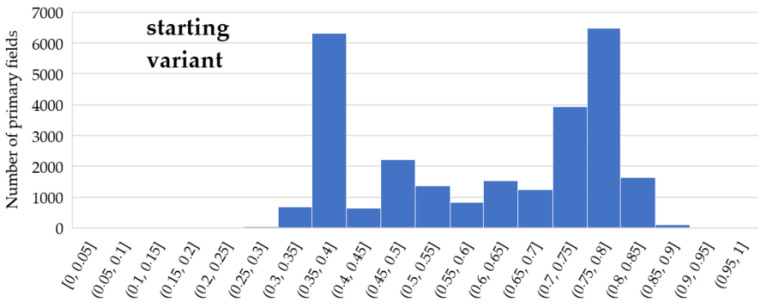
IOPs distribution in starting variant of passability map.

**Figure 17:**
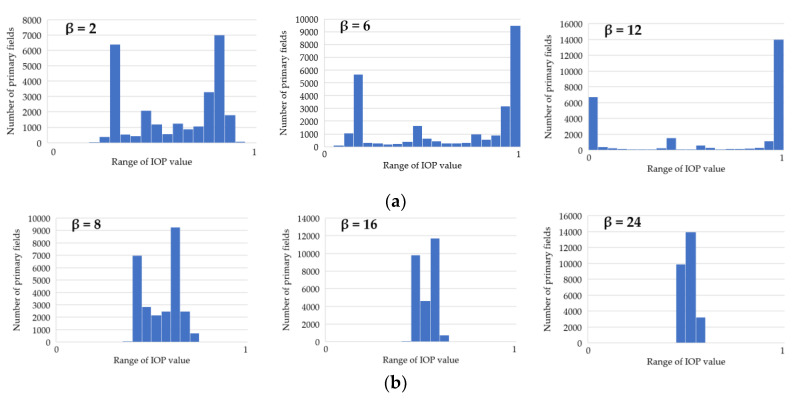
Distribution of IOPs on a test area in Warsaw: (**a**) Function (1)—stretch of IOP values; (**b**) Function (2)—aggregation of IOP values.

Moreover, the application of functions (1) and (2), which modify IOP values, enabled us to generate different variants of routes. The use of function (1) results in a route that is in most cases longer than the starting variant and crosses terrain that is easier to overcome. On the other hand, function (2) makes the routes shorter but much harder to overcome. All variants are presented in Figure 12, Figure 13 and Figure 14, where the resulting routes clearly prove that with the increase of β coefficient with use of function (1) the obtained route usually becomes longer. It is noticeable that its course avoids the areas of worse passability. Along with the rise of the β coefficient, the route moves to areas that are easier to overcome, somehow avoiding impassable areas with a wider and wider curve. The reason for that is the stretch of IOPs distribution resulting in an augmentation of differences between them. In practice, this means that lower indexes of passability (from 0 to 0.5) become progressively lower, while indexes greater than 0.5 increase and become close to 1. The use of function (2) has an opposite effect—the determined route becomes shorter with the increase of β coefficient. Its shape is increasingly closer to the straight line between two points which connects the start and end points in the shortest way. The direct reason for that is an aggregation of IOPs distribution, which practically makes the differences between obtained indexes of passability smaller. These dependences are presented in Figure 16 and Figure 17.

**Figure 18:**
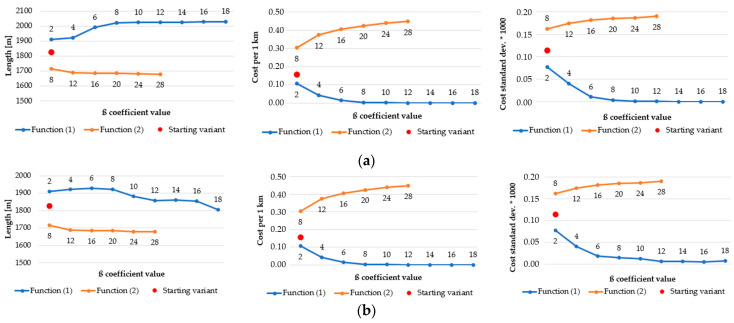
Area: urban, resolution of the passability map: 2 m, algorithm: (**a**) Dijkstra’s; (**b**) A-star.

**Figure 19:**
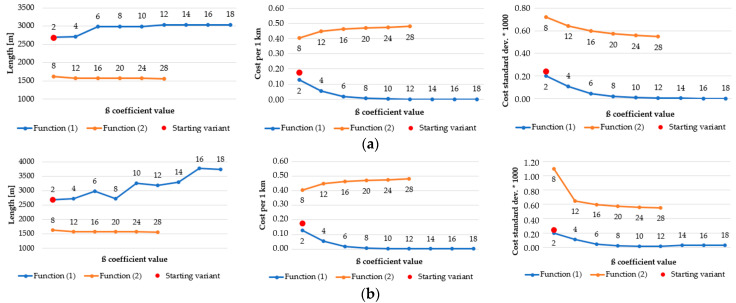
Area: undeveloped, resolution of the passability map: 5 m, algorithm: (**a**) Dijkstra’s; (**b**) A-star.

**Figure 20:**
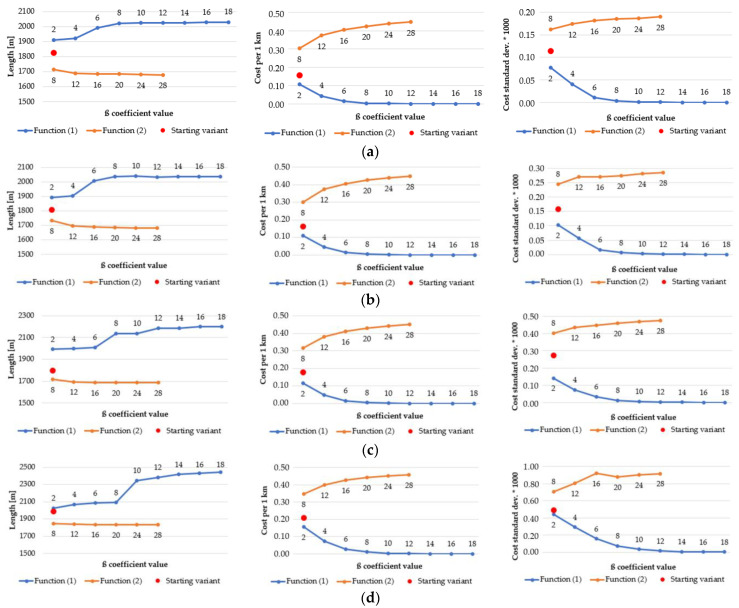
Area: urban, algorithm: Dijkstra’s, resolution of the passability map: (**a**) 2 m; (**b**) 3 m; (**c**) 5 m; (**d**) 10 m.

**Figure 21:**
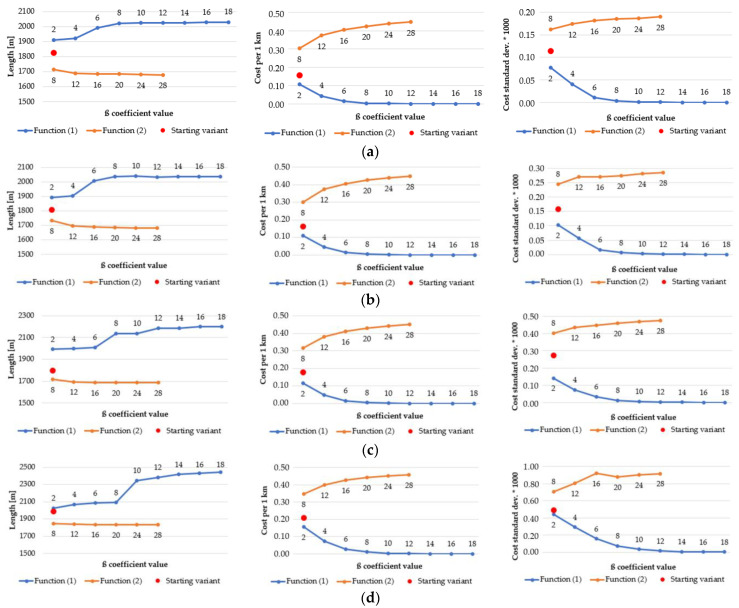
Area: undeveloped, algorithm: A-star, resolution of the passability map: (**a**) 2 m; (**b**) 3 m; (**c**) 5 m; (**d**) 10 m.

As a result of the application of various functions, algorithms, and β coefficients, the generated routes have various statistics. The most representative ones, which best reflect the nature of the route, are shown in Figure 18, Figure 19, Figure 20 and Figure 21. They present how the length of route and an estimator, which measures the “difficulty” of overcoming the route (average cost of overcoming 1 km of designated route), influence the change of β coefficient. In order to determine how the average cost of getting across each primary field changes, the standard deviation of the costs of overcoming the subsequent primary fields, followed by the route, was calculated. Charts are grouped in order to facilitate their analysis.

**Figure 22:**
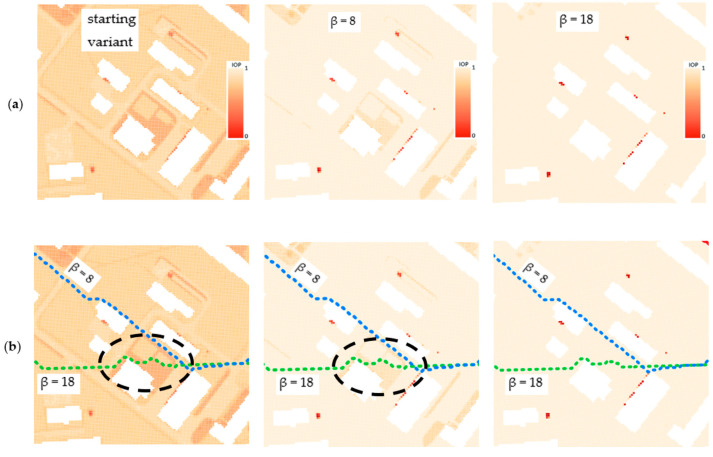
(**a**) Visualization of a passability map depending on β coefficient in function (1); (**b**) Resulting routes from function (1) of given β coefficient.

High standard deviation demonstrates that the variability of passability conditions is great and occurs when function (2) is used. This makes the routes shorter and more difficult to overcome (the cost increases with the growth of the β coefficient). If function (1) is applied, the route, despite being longer, passes through the primary fields whose indexes of passability change slightly and whose passability is good or very good. Reversely to Dijkstra’s algorithm, in A-star the length of routes generated with use of function (1) decreases with the growth of β coefficient. Dijkstra’s algorithm tries in this case to avoid obstacles or areas that are harder to overcome by taking a wider and wider curve, while the A-star algorithm tries to find the shortest way that passes through the primary fields of reduced IOP values (result of function (1)). This behavior will be explained based on Figure 22.

One may clearly notice that function (1) amplifies the differences between IOP values and using greater and greater β coefficient results in a passability map that consists of primary fields of values close to 0 and 1. IOPs from 0 to 0.5 in the starting variant are converted into values close to 0 and IOPs from 0.5 to 1 into values closer to 1, which is visible in Figure 22a. As far as the course of the routes is concerned (Figure 22b), one may notice that the green dashed route, which was created on the basis of passability map of β = 18, crosses terrain that has worse passability on passability maps resulting from lower values of β coefficient (in case of Figure 22—β = 8) or from the starting variant. The expression ‘worse passability’ refers to a terrain which has lower IOP values, and consequently it is marked in more saturated color on the passability map. Such situations occur in area surrounded by a black dashed line in Figure 22. However, in the passability map of β = 18, which is corresponding to the designated green dashed route, the route does not cross the worse passable area.

In conclusion, the use of function (1) blurred the differences between IOP values from 0 to 0.5 and from 0.5 to 1. The A-star algorithm was searching for the shortest route in the end point direction and consequently, in greater β values it managed to find a route that was shorter than the previous ones and had a lower cost as well. This was due to the fact that the areas of slightly worse passability were converted with use of a function (1) into areas of a good passability. As a result, the A-star algorithm cannot be treated as fully secure, because for greater values of β coefficient in function (1) it can generate a route that leads through areas which actually have worse passability. In Dijkstra’s algorithm this situation never happens, and it avoids all obstacles with an increasingly wide curve, which makes the resulting route more secure.

Figure 19 also shows the comparison of the two discussed algorithms, but this time for a higher resolution (5 m). Here, one may observe the same dependences in both outcomes. The length of routes in function (1) grows with the increase of β coefficient but in case of A-star algorithm the growth is not as steady as in Dijkstra’s (Figure 19b). The biggest fluctuation is in β = 8, where the length of a route unexpectedly drops. The rest of the statistics behave quite similarly.

As far as the Dijkstra’s algorithm in different resolutions is concerned (Figure 20), all statistics have the same pattern. In function (1) the length of the routes grows, but the cost per 1 km and the cost standard deviation decrease steadily. The inverse situation may be observed in case of function (2). It is worth noticing that the generated routes become longer along with the growth of the pixel size of a passability map, e.g., in the 2 m resolution map the longest route is slightly over 2000 m long but in the 10 m resolution map the length of the longest route increases to approximately 2450 m. The standard deviation values also grow with the increase of the resolution. However, the statistics from Dijkstra’s algorithm seem to be more stable than those from A-star, which is clearly visible in Figure 21.

As described earlier, in this situation the length of the routes decreases with the increase of the β coefficient (2 m and 3 m resolution). In 5 m and 10 m maps, the length behaves similarly to Dijkstra’s algorithm (it is growing). Moreover, the cost per 1 km value is almost the same in each case but the standard deviation of costs is diversified. All its values become greater with the increase of the resolution, which proclaims that in lower resolutions the variability of passability conditions is more significant.

**Figure 23:**
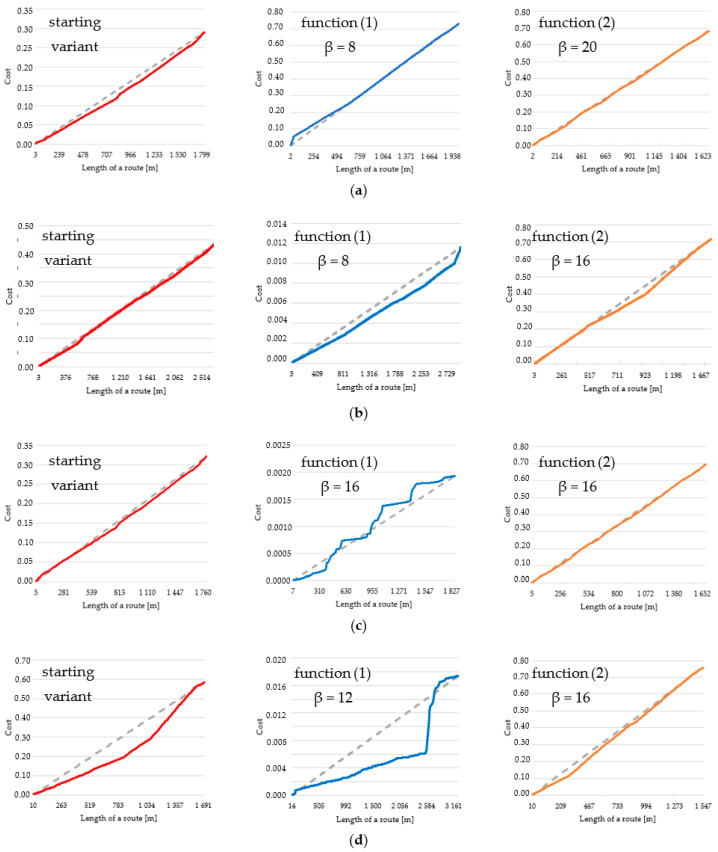
Cumulative cost charts of the following routes: (**a**) resolution: 2 m, algorithm: Dijkstra’s, area: urban; (**b**) resolution: 3 m, algorithm: Dijkstra’s, area: undeveloped; (**c**) resolution: 5 m, algorithm: A-star, area: urban; (**d**) resolution: 10 m, algorithm: A-star, area: undeveloped.

What is more, the cumulative cost charts, which are presented in Figure 23, offer other interesting results. They show the change of cost with the increase of the distance from start to end point. Charts were created for three variants of routes in four different situations (various parameters). The grey dashed lines mark the growth of the cost when it is stable and equal along the whole route, while the colored lines signify real values.

**Figure 24:**
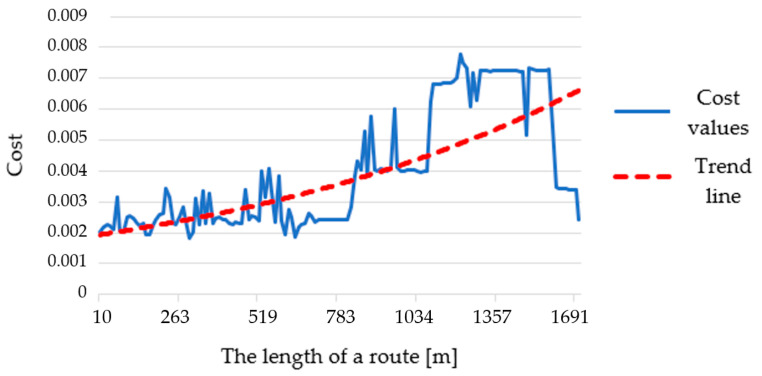
Cost of edges chosen by the algorithm in relation to the length of a route on the example of starting variant of the route—resolution: 10 m, algorithm: A-star, area: undeveloped.

Various β coefficients of routes, which were the basis for creating the charts, were adjusted in order to show the changes between them and starting variant as clearly as possible. On the cumulative cost charts, one may see mainly that in most cases the routes are designated on the basis of edges of the same or close costs (the colored and dashed lines overlap largely). Such situation is visible in all cases of function (2), in the starting variant without the lowest resolution case (Figure 23d) and in function (1) in the highest resolution case (Figure 23a). The remaining charts are not as stable, and the highest volatility may be observed for function (1) in 5 m and 10 m resolution cases (Figure 23c,d). However, in most cases the cost in the early parts of the routes is smaller than the average value (the colored line is below the dashed line) and in the closing parts the cost becomes higher. It makes the charts concave in relation to the average value (grey dashed line). It means that the used algorithms mainly generate routes with gradually increasing cost. An example of that is shown in Figure 24.

One may easily notice that the trend line of costs is upward, which means that the further away we are moving, the higher the difficulty of moving along the designated route is becoming. Such a situation is present in most obtained results.

As far as the performance of the methodology is concerned (Table 3), the generation time of one route decreases due to the growth in the size of a primary field of the passability map used. The bigger the primary field is, the fewer edges the algorithm has to process to obtain a route between two points. It certainly has the biggest influence on the route generation time. It is also worth noting that in this research project, the routes were only about 2 km long and if the start and end points were much further apart, the generation of routes would also take much longer. Moreover, there is a slight difference between Dijkstra’s and A-star algorithms. Despite the fact that the A-star algorithm needs to analyze fewer edges to find an optimal route (Figure 9), it works a little bit slower than Dijkstra’s. The comparison of the route determination times in different resolutions reveals that the A-star algorithm needs about 20–25% more time to determine the optimal route. With the increase of a distance between start and end point the route determination time in Dijkstra’s algorithm can be significantly shorter. What is more, the longest part of the route generation process is the export of the designated route to an SHP file. It takes about 95% of the whole process. This is caused by the queries that are sent to the database (PostgreSQL in case of this research) in order to create a separate file containing selected graph edges. One may see that the SHP generation times in Dijkstra’s and A-star algorithm are more or less the same, which means that this part of the process is independent from the used algorithm.

## 5. Conclusions

The methodology proposed in this article shows the way to obtain a graph that allows one to find the optimal route, on the basis of the developed passability map built of square primary fields. Due to the fact that the developed passability maps take into account most land cover elements, the generated route is credible and thanks to the use of Dijkstra’s or A-star algorithm it can be treated as optimal. It turned out that modifying the indexes of passability, which are the basis for generating graphs, and consequently routes, was an interesting experiment. The use of function (1) allowed us to obtain a route that was usually longer, but crossing well passable areas. Routes generated with the use of function (2) were significantly shorter but they led through areas of worse passability. Such variant approach improves the possibility of planning the movement of troops or the determination of, e.g., UGV movement.

The detection of impassable areas allows users to remove edges that intersect them from the graph. This operation ensures that the designated route will not cross any areas that are impossible to overcome. The authors assumed that impassable areas are buildings and terrain with microrelief shapes that the vehicle may get stuck on. The methodology of detecting impassable areas due to the landform is presented in article [29].

The obtained results demonstrate that the change of the resolution of a passability map affects the courses of generated routes, as it was presented in Figure 15. The reason for that is the generalization of the passability map. In higher resolutions the routes in some cases may maneuver between dispersed impassable places while in lower resolution the degree of generalization is so big that the routes tend to completely avoid clusters of these areas (Figure 15, 1).

The change of the β coefficient in used functions allowed us to obtain different variants of the generated routes and to see how they behave for various β values. As it was expected, in most cases the routes become longer while increasing the β coefficient in function (1) and the routes become shorter with the increase of this parameter in function (2). However, in some cases the output routes do not follow this rule and their length decreases while augmenting β coefficient. This behavior happens only in the A-star algorithm and the reason of that has been described on the basis of Figure 19 in the previous section. This makes the Dijkstra’s algorithm more secure because it always avoids areas that are harder to overcome. For greater β values in function (1), it never generates a route that crosses terrain of an actually worse passability. On the other hand, in very detailed passability maps (2 m and 3 m) in greater β values in function (1) the A-star algorithm generated routes that led through less passable areas. In this algorithm it is sufficient to set a small β value (not exceeding 8) in function (1) to obtain a route that meets the assumptions of this variant—longer but easier to overcome. Due to the fact that we do not know the exact value of β, which will cause the shortening of a route and lead it through areas that are harder to overcome, it is more secure to use Dijkstra’s algorithm. 

The cumulative cost charts also provide a good overview of the characteristics of the generated routes. From them (Figure 23) one may conclude that the distribution of costs along the route was mainly stable. The cost line was in most cases close to a straight line connecting 0 and total cost value of a route. However, the A-star algorithm turned out to be not as stable as Dijkstra’s. Taking into account parts (c) and (d), one may see in function (1) a high volatility of cost along the route. What is more, cost lines are mainly located below the average cost value line (grey dashed line), which means that the cost in initial parts of a route is lower and in further part there is a visible increase. This is confirmed by the chart presenting a trend line of the chosen route (Figure 24), where it is visibly growing. Such a situation happens for most of the generated routes.

Moreover, according to Table 3, which presents the results of the performance analysis, the generation time of the routes decreases due to the increase of a primary field size of the passability map. It has the biggest influence on generation time, and it happens because in lower map resolution the graph consists of fewer edges than in maps of higher resolution. As far as the used algorithms are concerned, despite having more primary fields to analyze, Dijkstra’s algorithm is about 20% faster than A-star. A major part of the route generation time is the SHP file generation, which takes about 95% of the whole duration of the process. It is caused by the queries that are sent to the database in order to extract the selected edges and create a new file consisting only of a single route.

The presented methodology is universal because it can be used for planning the movement in a wide maneuver corridor (1 km) as well as for the several-meter corridor on the basis of detailed maps of passability. Analyzes showed that Dijkstra’s algorithm is more convenient to use than A-star because it is more stable and works faster. It is worth mentioning that the module of route generation is an integral part of an IT system, which provides fully automated development of passability maps, their conversion into graphs, and also automated route generation. The area of application of the constructed system is very wide because, except for planning the movement of large military units or UGVs, the use of detailed passability maps is of key importance for crisis management, where the possibility of reaching the area outside the road network can be crucial for a success of the salvage operation.

## Figures and Tables

**Table 1:** Relation between troop size and size of primary field.

Troop Size	Troop Symbol	Size of Primary Field	Width of an Avenue of Approach
Single vehicle	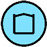	2–5 m	10 m
Team	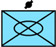	10 m	20 m
Squad	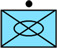	20 m	50 m
Section	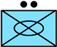	50 m	100 m
Platoon	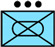	100 m	200 m
Company	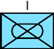	200 m	500 m
Battalion	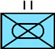	500 m	1500 m
Brigade	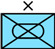	1000 m	3000 m
Division	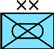	2000 m	6000 m
Corps	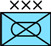	5000 m	15,000 m

**Table 2:** Examples of passability maps in different resolutions.

**200 × 200 m**	**1000 × 1000 m**	**2000 × 2000 m**
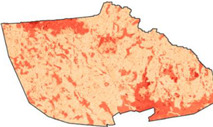	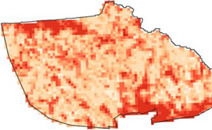	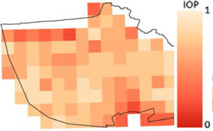
**3 × 3 m**	**10 × 10 m**	**20 × 20 m**
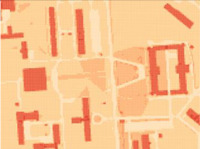	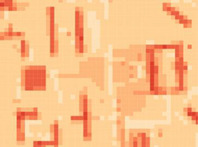	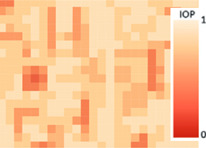

**Table 3:** Generation times of a starting variant route in undeveloped area.

Resolution	Generation Times					
	Dijkstra’s Algorithm			A-Star Algorithm		
	Route Determination	Export to SHP File	Total Time	Route Determination	Export to SHP File	Total Time
2 m	7.46 s	2 min 52.95 s	3 min 0.41 s	8.91 s	2 min 48.74 s	2 min 57.65 s
3 m	2.90 s	1 min 14.40 s	1 min 17.30 s	3.57 s	1 min 14.47 s	1 min 18.04 s
5 m	0.86 s	32.42 s	33.28 s	1.05 s	32.88 s	33.93 s
10 m	0.27 s	4.56 s	4.83 s	0.34 s	4.52 s	4.86 s
